# Vascular endothelial growth factor‐C in activating vascular endothelial growth factor receptor‐3 and chemokine receptor‐4 in melanoma adhesion

**DOI:** 10.1111/jcmm.17571

**Published:** 2022-11-17

**Authors:** Yvette N. Hlophe, Anna M. Joubert

**Affiliations:** ^1^ Department of Physiology University of Pretoria Pretoria South Africa

## Abstract

Vascular endothelial growth factor‐C (VEGF‐C) binds to receptor vascular endothelial growth factor receptor‐3 (VEGFR‐3) expressed on lymphatic endothelial and melanoma cells. Binding of VEGF‐C to VEGFR‐3 enhances receptor phosphorylation that activates mitogen‐activated protein kinase (MAP‐K) and phosphatidylinositol‐3‐kinase (PI3K). These signalling pathways regulate cell migration and adhesion in response to internal or external changes.

In addition, the overexpression of VEGF‐C upregulates chemokine receptor CXCR‐4 in tumours (melanoma). CXCR‐4 is expressed on cells of the immune system (natural killer cells) and facilitates the migration of leukocytes in response to the CXCL12 ligand. The latter is expressed by lymphatic endothelial cells and by stromal cells in the tumour microenvironment (TME). The gradient established between CXCR‐4 expressed on tumour cells and CXCL12 produced by stromal and lymphatic endothelial cells enhances tumour cell metastasis.

3‐(4‐Dimethylamino‐naphthalen‐1‐ylmethylene)‐1, 3‐dihydroindol‐2‐one, MAZ‐51, is an indolinone‐based synthetic molecule that inhibits the phosphorylation of the tyrosine kinase receptor VEGFR‐3. CTCE‐9908, a CXCR‐4 antagonist derived from human CXCL12, hinders receptor phosphorylation and the subsequent signalling pathways that would be activated.

VEGF‐C is stimulated by transforming growth factor‐beta 1 (TGF‐β1), which facilitates cell–cell and cell‐matrix adhesion by regulating cadherins through the activation of focal adhesion kinase (FAK) and mediates paxillin upregulation.

Increased VEGF‐C protein levels stimulated by TGF‐β bound to VEGFR‐3 impact on intracellular pathways that promote tumour cell adhesion. In addition, increased VEGF‐C protein levels lead to enhanced CXCR‐4 protein expression. Therefore, effective blocking of VEGR‐3 and CXCR‐4 may inhibit tumour cell metastasis by hampering intracellular proteins promoting adhesion.

## 1 INTRODUCTION

### 1.1 Epidemiology

Melanoma is a cancer of the skin melanocytes responsible for producing skin pigmentation.^1^ This aggressive malignancy increases the chances of metastasis from the primary site of the tumour.^2^ Due to the latter, the survival rate with current therapeutics is less than 5 years depending on the progression of the melanoma at diagnosis.^2^ The National Cancer Institute (NCI) estimates 106,110 new melanoma cases accompanied by 7180 melanoma deaths for 2021.^3^ In addition, and based on 2016–2018 data, 2.3% of men and women will be diagnosed with melanoma of the skin in their lifetime.^3^

Skin cancer is categorized into three subdivisions namely melanoma, squamous cell carcinoma (SCC) and basal cell carcinoma (BCC). SCC and BCC are referred to as non‐melanoma skin cancer (NMSC).^4^ Melanoma is a relentless form of cancer since it accounts for 1%–2% of all cancer mortalities globally.^2^ The most common skin cancer is the non‐melanoma skin cancer of, which 80% are affiliated with BCCs.^5^ Metastatic BCCs are not common (0.0028%–0.55%), although the occurrence of BCC incidences is increasing.^6^ Patients with metastatic BCCs have a five‐year survival rate of 10%. However, the ten‐year survival rate of these patients declines below 10%.^7^

Non‐melanoma skin cancer including BCC and SCC impact more than 3 million Americans annually.^8^ It has been predicted that 196,060 new cases of melanoma, 101,280 noninvasive (in situ) and 106,110 invasive cases will be diagnosed in 2021 in the US.^8^

The melanoma staging system was initially established by the American Joint Committee on Cancer (AJCC) Melanoma in 2001 but has been revised eight times. This staging system is used to categorize criteria to determine each stage.^9^ Patients categorized in stage I or II refer to melanoma that has not metastasised to local or distant sites. In stage III patients, melanoma is detected in regional lymph nodes and intra‐lymphatic sites. Stage IV indicates melanoma metastasis to distant sites.^2^ The prognosis of patients with stage IV melanoma is based on the secondary site where the cancer has metastasised.^9^ In stage IV melanoma patients, if the cancer has metastasised to the skin in areas further away from the primary site, or is located in lymph nodes, this decreases the percentage determined for a 1‐year survival rate. The latter indicates that melanoma metastasis is a significant predictor of melanoma prognosis.^2^ Migration, a metastatic characteristic of tumour cells allows for movement from the primary mass towards blood or lymphatic vasculature permitting the transport of the tumour cells towards a secondary site.

### 1.2 Tumour microenvironment in melanoma metastasis

Healthy cells in the tumour microenvironment provide the growth factors and cytokines required by the tumour cells and the extracellular membrane (ECM) regulates biochemical activity in the microenvironment.^10^ ECM molecules bind integrin receptors on the cell membrane facilitated by growth factors referred to as ‘inside‐outside signalling’.^11^ The integrin ECM growth factor activity promotes the signal transduction protein RAS, which leads to RAS^12^ activation in the intracellular membrane^10^; RAS proteins form part of a superfamily of guanosine triphosphate **(**GTP) binding proteins, which regulate signal transduction.^12^ The ECM coordinates cell adhesion molecules that bind to cytokines/chemokines and growth factors.^13^ The integrin‐mediated effectors, focal adhesion kinase (FAK) and Src are non‐tyrosine kinases associated with downstream signalling of mitogen‐activated protein kinase (MAP‐K), Rac1 and GTPases are frequently studied in cancer.^14^

An increased collagen matrix in tumour microenvironment (TME) enhances tumour cell adhesion and therefore contributes to melanoma metastasis.^15^ Melanoma cells have metastatic characteristics activated by signalling pathways that are activated by receptor phosphorylation induced by growth factors (vascular endothelial growth factor‐C (VEGF‐C) and transforming growth factor‐beta (TGF‐β1)). An increase in protein levels of VEGF‐C is stimulated by other growth factors including TGF‐β^16^ thereby contributing to metastasis of the tumour cells.

Melanoma cells follow several steps to metastasise from the primary tumour to the secondary site. These steps include (i) tumour cells infiltrating the surrounding tissue, (ii) cancer cells migrating until they intravasate into vasculature, (iii) tumour cells have to be sustained as they travel in the circulatory system, (iv) extravasation out of the vasculature, (v) tumour cells migrating to secondary tissue, adherence to the basement membrane and proliferation at the secondary site.^17^

## 2 GROWTH FACTOR RECEPTORS AND LIGANDS

### 2.1 Vascular endothelial growth factor family

Growth of endothelial cells (ECs) found in arteries, veins and lymphatics are promoted by VEGF, which inhibits apoptosis and promotes fenestrations of ECs and vascular permeability.^18^ VEGF levels are enhanced by cytokines and growth factors such as fibroblast growth factor (FGF), platelet‐derived growth factor (PDGF) and transforming growth factor (TGF).^18^

The cellular function of vascular endothelial growth factor (VEGF) family (VEGF‐A, B, C, D E) and placental growth (PIGF)^19^ includes the pro‐angiogenic role of VEGFs, which sustains the vasculature of several tissues^20^; VEGF‐A is known to maintain vascular permeability and endothelial cell migration. VEGF‐B prevents the degeneration of sensory neurons. VEGF‐C is highly expressed during embryogenesis and VEGF‐D is expressed postbirthing into adult stages.^20^ VEGF‐C and VEGF‐D are strongly affiliated with lymphatic vasculature and promote lymphangiogenesis.^20^ VEGF‐E is coded in the Orf virus genome and has a strong affinity to VEGFR‐2 in comparison to the other growth factor ligands.^20^ PIGF only binds VEGFR‐1 and therefore promotes angiogenesis.^20^ The latter ligands are similar in structure and bind to specific tyrosine kinase receptors (VEGFR‐1, 2 or 3) with neuropilin co‐receptors (NRP 1 and NRP 2).^21^ VEGF‐C and VEGF‐D bind VEGFR‐2 and ‐3, respectively; however, the ligands are affiliated to lymphangiogenesis, which is associated with VEGFR‐3.^21^ Research has shown that, in mice, VEGF‐D only binds to the lymphangiogenic receptor VEGFR‐3 and not the angiogenic receptor VEGFR‐2.^22^ PIGF binds VEGFR‐1 during angiogenesis of pathological states, and VEGF‐A, the most frequently studied growth factor, binds to VEGF‐1 in instances of angiogenic activity.^23^ This assists to maintain blood vasculatures that sustain tissue function, but in pathological states, this can sustain tumour growth.

### 2.2 Vascular endothelial growth factor‐C

Neoplastic cells expressing high levels of VEGF‐C have been associated with tumour dissemination through the lymphatic vasculature.^24^ Literature has shown a link between high VEGF‐C protein levels in melanoma located in the primary tissue and the activity of lymph node metastasis by means of VEGFR‐3/VEGF‐C a gradient.^24^ The latter indicates that melanoma cells migrate to lymph nodes from primary sites through lymphatic vasculature.

The increased VEGF‐C levels are associated with a low survival rate in lung, oesophageal squamous and the lymph nodes of metastatic melanoma patients.^25^ VEGF‐C hinders the effect of phosphorylate phospholipase‐C on the anti‐tumour immune responses, therefore enhancing melanoma metastasis.^26^

### 2.3 Transforming growth factor‐beta 1

TGF‐β has three isoforms,^1^, ^2^, ^3^ which are produced within the cell as dimeric prohormones.^27^ The inactive form of TGF‐β moves to the extracellular environment where it is cleaved to convertase and furins into the active form of TGF‐β.^27^ Signalling by TGF‐β occurs when the active ligand binds two pairs of receptors serine/threonine kinases (receptor I and II) composing the heterometric structure.^27^ Once receptor phosphorylation has occurred, TGF‐β pathways are activated.^27^ TGF‐β1 inhibits growth in healthy epithelial cells and melanocytes.^28^

Melanomas are able to resist the inhibitory effects of TGF‐β1.^28^ TGF‐β1 contributes to the progression of melanomas as melanoma growth correlates with increasing TGF‐β1.^28^, ^29^ The TGF‐β1 in melanoma causes fibroblast to stimulate the matrix around the tumour mass.^28^ Increased collagen and fibronectin matrixes were detected in melanomas with high TGF‐β1 protein levels^28^ indicating the role of TGF‐β1 in melanoma adhesion.

**FIGURE 1:**
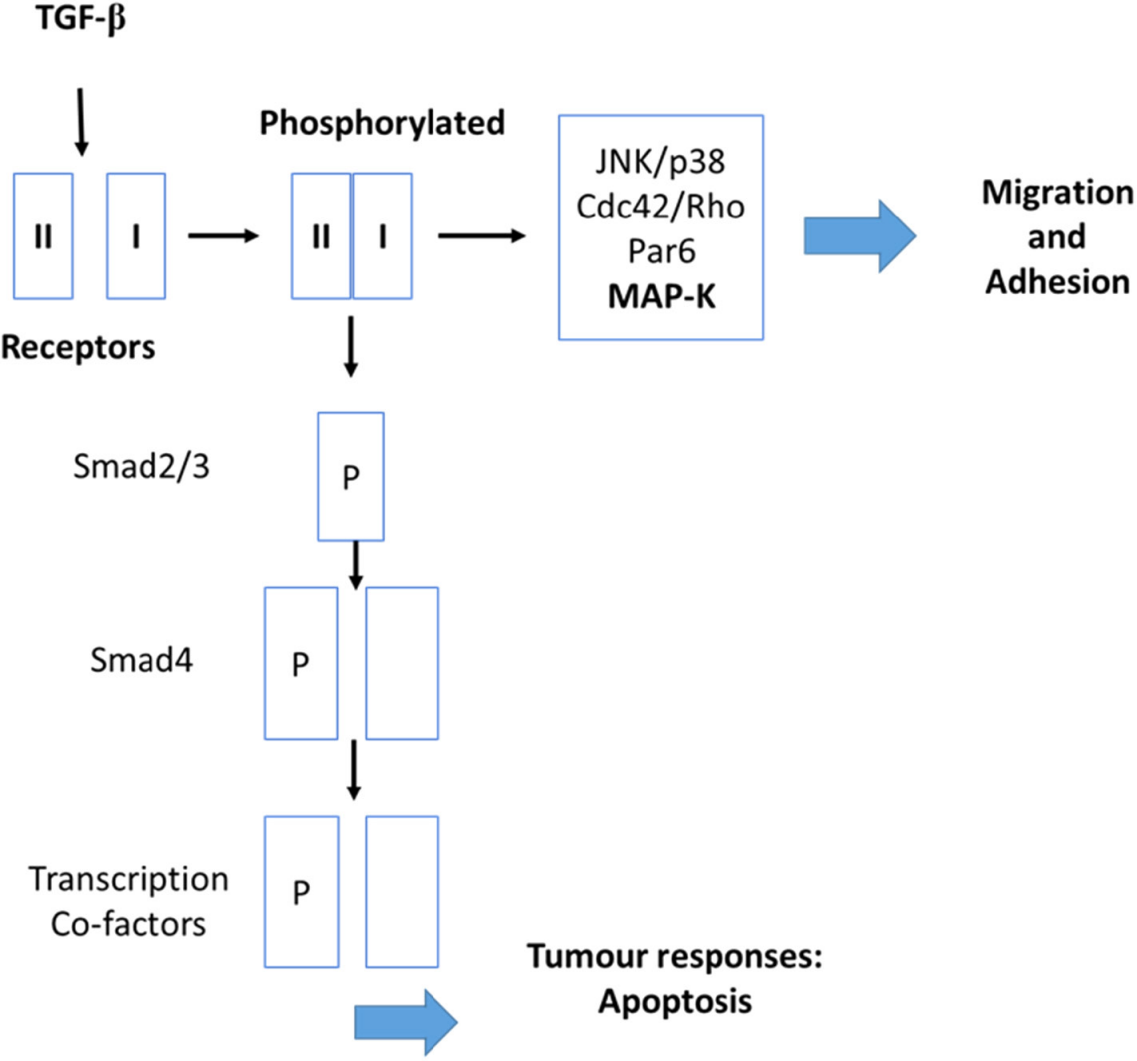
TGF‐β ligand binding the two pair serine/threonine receptor activating receptor phosphorylation and triggering the signalling pathways JNK/p38, Cdc42/Rho (family of GTPases) and Par6 affecting migration and adhesion, cell shape and cell‐to‐cell contact. Additional TGF‐β signalling may occur through the Smad pathway where TGF‐β gene transcription enhances tumour suppressive responses that enhance apoptosis.^27^ This indicates the dual role of TGF‐β to enhance tumour cell metastasis or to induce apoptosis depending on the intracellular pathway activated. (Image was designed by Y.N Hlophe using Microsoft PowerPoint 2013; 2013 Microsoft Corporation).

In the progress of melanoma development, tumour‐activated macrophages release TGF‐β1 and, in an autocrine manner, express VEGFR‐3 and secrete VEGF‐C and VEGF‐D thereby promoting lymphangiogenesis and thus lymphatic metastasis of melanoma cells.^30^ In melanoma cells, TGF‐β1 enhances melanoma growth and progression, while in melanocytes, TGF‐β1 has a growth inhibitory effect.^31^ Lack of sensitivity of the melanoma cells to the growth inhibitory effects of TGF‐β1 in comparison with melanocytes was noted.^31^ Human melanoma cells treated with exogenous TGF‐β1 increased concentrations of all three isoforms namely TGF‐β1, TGF‐β2 and TGF‐β3 demonstrating the autocrine functioning of TGF‐β in melanoma cells.^29^ The pathway of interest concerning TGF‐β is the c‐Jun N‐terminal kinases/protein 38 (JNK/p38) JNK/p38 MAP‐K, which regulates adhesion and migration (Figure 1).^27^

### 2.4 Vascular endothelial growth factor receptor‐3

The VEGFR‐3 extracellular binding domain comprises seven immunoglobulin‐like domains with a tyrosine kinase intracellular domain.^32^ VEGFR‐3 phosphorylation of the dimerised tyrosine kinase sites monitors kinase activity and the communication with signal transduction molecules such as JNK1/2, ERK 1/2 and Akt (Figure 6).^32^ Once VEGFR‐3 is phosphorylated, it activates signalling pathways that lead to cell migration (Figure 6).^32^ Singh et al identified a soluble isoform of VEGFR‐3 in epithelial cells of the cornea.^33^ Mimura et al were one of the groups to identify VEGFR‐3 on a nonendothelial ocular surface.^34^ Hamrah et al identified VEGFR‐3 on inflamed conjunctiva as monocyte‐derived bone marrow cells. VEGFR‐3 was also observed on inflamed corneal dendritic cells.^35^ The soluble VEGFR‐3 binds VEGF‐C and therefore inhibits VEGF‐C binding VEGFR‐3.^33^ In addition to their presence on ECs, receptors to VEGFs are expressed on haemopoietic stem cells, immune cells such as dendritic cells and cancer cells.^18^ *Wilting* et al were the first to report the presence of VEGFR‐3 on nonendothelial cells, namely the kidney glomeruli podocytes and quail embryos.^36^ Inflamed dendritic cells express VEGFR‐3 and VEGF‐C, but in noninflamed dendritic cells, the intracellular expression of VEGFR‐3 increases and VEGF‐C as it binds NRP‐2.^18^ The VEGFR‐3/VEGF‐C signalling is known to activate innate and adaptive immune responses.^18^

High VEGF‐C melanoma levels create a gradient via VEGFR‐2 expressed on blood endothelial cells and promote angiogenesis.^37^ Several studies have shown that tumours overexpressing VEGF‐C tend to promote metastasis of the tumour cells^38^ far more than promoting tumour growth.^26^, ^39^, ^40^

Endotheliomas originating from blood endothelial cells express vascular endothelial growth factor receptor (VEGFR‐3).^41^ Partanen et al have shown that VEGFR‐3 is found in benign and malignant tumours of vascular origin.^42^ The upregulation of VEGFR‐3 on blood endothelial cells is due to the presence of its ligand, VEGF‐C, in the vascular tumours.^43^ In addition, VEGF‐C is also an active ligand of the blood endothelial receptor, VEGFR‐2, that plays a key role in angiogenesis.^43^ Since VEGFR‐3 protein levels were detected in blood endothelial cells in healthy tissue^39^, ^43^, ^44^ it is suggested that tumours expressing VEGFR‐3 still metastasise predominantly through the lymphatic vasculature. They can, however, create a VEGFR‐3/VEGF‐C gradient with blood endothelial cells and therefore metastasise through blood and lymphatic vasculature. This is contrary to the previous understanding that VEGFR‐3 was receptor‐specific to lymphatic endothelial cells.^45^ The latter implies that tumours previously thought to only metastasise through the lymphatic vasculature due to VEGFR‐3 expression, also have the possibility of metastasising through the blood and lymphatic vasculature.

#### 2.4.1 Vascular endothelial growth factor receptor‐3/vascular endothelial growth factor‐C gradient

The VEGFR‐3/VEGF‐C gradient is established when the VEGFR‐3 expressed on tumour cells creates a pulling factor for the tumour cells towards the lymphatic endothelial cells expressing VEGF‐C.^46^ The gradient established by VEGFR‐3 and VEGF‐C enhances cancer cell migration and the ability of the cells to invade the lymphatics.^47^ Su, Jen Liang et al were able to demonstrate that the VEGFR‐3/VEGF‐C autocrine loop was defective in the human lung carcinoma cell line with a deleted VEGFR‐3 (A549 cells).^47^ The VEGFR‐3/VEGF‐C gradient has shown to enhance tumour cell proliferation, survival and migration in Kaposi sarcoma cells, malignant mesothelioma cells, leukaemia cells, lung adenocarcinoma, cervical and prostate cancer.^40^ Tumour cells expressing VEGFR‐3 are also known to secrete VEGF‐C suggesting that the mechanism of operation can be an autocrine/paracrine manner between receptor and ligand.^40^

In human solid tumours, the expression of CXCR‐4 and VEGF are measurable predictors of tumour metastasis^48^ since expression levels increase in solid tumours. The CXCR‐4/CXCL12 axis regulates the adhesion of haematopoietic cells expressing CXCR‐4 and endothelial and stromal cells secreting CXCL12. VEGF in the bone marrow environment promotes the expression of CXCR‐4 thereby enhancing the chemotaxis of the haematopoietic cells.^49^ *Zhuo* et al indicated that CXCR‐4 expression is upregulated by VEGF‐C in lymphatic endothelial cells (LECs).^50^

### 2.5 C‐X‐C motif chemokine receptor‐4

Chemokines are proteins that function as chemoattractants for immune cells.^51^ With more than 50 members of the G‐protein‐coupled receptors associated with chemokines, there is an additional division into four sub‐families. C‐X‐C motif chemokine receptor‐4 (CXCR‐4) is a well‐known member of the chemokine receptor family, because of the multiple roles it plays in the immune response, tumorigenesis, developmental processes and haematopoiesis.^52^ The expression of CXCR‐4 is not isolated to haematopoietic cells.^51^ The receptor binds the glycoprotein cluster of differentiation‐4 (CD4), the transmembrane protein CD47 and CXCL12 the stromal‐derived factor 1α (SDF‐1α), which is specific to CXCR‐4.^51^ The ligand CXCL12 binds an additional receptor CXCR‐7 and is secreted in nonhaematopoietic tissue such as the brain, lungs and stromal endothelial cells and bone marrow, where it attracts haematopoietic stem cells expressing CXCR‐4.^52^

The CXCR‐4 receptor is the most prevalent chemokine receptor expressed by melanoma cells.^53^ B‐16 melanoma cells expressing CXCR‐4 binding to its corresponding ligand, CXCL12,^54^ expressed by lymphatic endothelial cells enhanced B‐16 melanoma adhesion to the lymphatic endothelial cells thereby enhancing B‐16 metastasis.^54^ CXCL12 expressed by stromal cells^55^ and lymphatic endothelial cells^56^ bind specifically to receptor CXCR‐4, which stimulates intracellular signalling pathways MAP‐K and phosphatidylinositol 3‐kinase (PI3K), which are prevalent downstream pathways^57^ involved in cell survival, adhesion, proliferation and migration^58^ (Figure 3).

CXCR‐4 is stimulated by coupling of the intracellular heterotrimeric G‐protein linked to the intracellular portion of the plasma membrane.^58^ The subunits that comprise the heterotrimer include G‐beta (Gβ), G‐alpha (Gα) and G‐gamma (Gγ) bind to guanine dinucleotide phosphate (GDP).^58^ Ligand binding to CXCR‐4 replaces GDP with guanosine‐5′‐triphosphate (GTP) resulting in subunit dissociation, a βγ unit and an α unit to, which the GTP binds.^58^ The GTP then hydrolyses to GDP and the G‐protein heterotrimer is restored.^58^ The Gα subunit activates adenylate cyclase and MAP‐K signalling.^58^

Melanoma cells also express CXCR‐4, a chemokine receptor,^59^ with CXCL12 as a corresponding ligand.^60^ The CXCR‐4/CXCL12 gradient activates intracellular signalling pathways such as MAP‐K and PI3K^61^ that promote melanoma survival proliferation, migration^62^ and adhesion (Figure 3).

## 3 RECEPTOR ANTAGONISTS

In melanomas, the use of VEGFR‐3 inhibitors has proven to reduce metastasis to lymph node sites.^2^ The use of VEGF‐C and VEGF‐D, as well as VEGFR‐3 inhibitors,^63^ has also been tried to block signalling pathways. However, the use of receptor and ligand inhibitors was not proven successful to inhibit metastasis entirely in the clinical trials conducted^64^ ^.^ ^65^

### 3.1 Inhibition of receptor 3 phosphorylation by (3‐(4‐Dimethylamino‐naphthalen‐1‐ylmethylene)‐1, 3‐dihydroindol‐2‐one)

**FIGURE 2:**
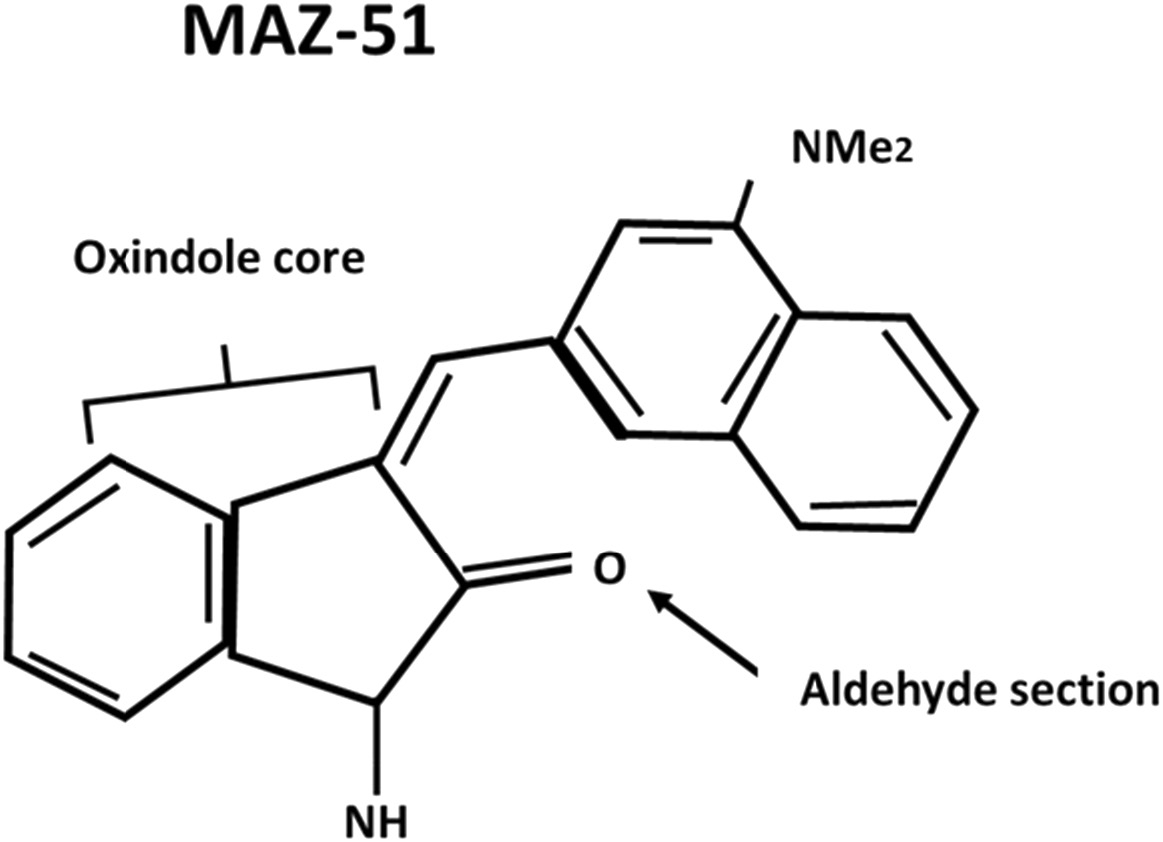
Chemical structure of MAZ‐51. An indoline at the oxindole core binds the adenine ring of the vascular endothelial growth factor receptor‐3.^66^ The aldehyde section determines the tyrosine kinase receptor that MAZ‐51 can bind to.^70^ (Image was designed by Y.N Hlophe using Microsoft PowerPoint 2013; 2013 Microsoft Corporation).

MAZ‐51 is an indolinone‐based synthetic molecule (Figure 2) that inhibits the phosphorylation of the tyrosine kinase receptor VEGFR‐3.^66^ Indolinones are part of a class of adenosine triphosphate (ATP) competitive receptor tyrosine kinase inhibitors that inhibit VEGF.^67^, ^68^, ^69^ Indolinone derivatives exert antiproliferative activity in cancer cell lines indicating their potential to inhibit proliferation and induce apoptosis in cancer cells..^46^, ^67^, ^70^, ^71^, ^72^ Phosphorylation of VEGFR‐3 is crucial in the lymphangiogenic process. The inhibitor, MAZ‐51, limits cellular proliferation in endothelial^73^ and cancer cells and activates apoptosis in cancer cells.^74^ MAZ‐51 has the ability to inhibit tyrosine kinase activity and therefore inhibits VEGF‐C binding to VEGFR‐3 therefore hindering cellular activity such as proliferation and lymphangiogenesis.^73^

### 3.2 Antagonist of CXCR‐4 CTCE‐9908

**FIGURE 3:**
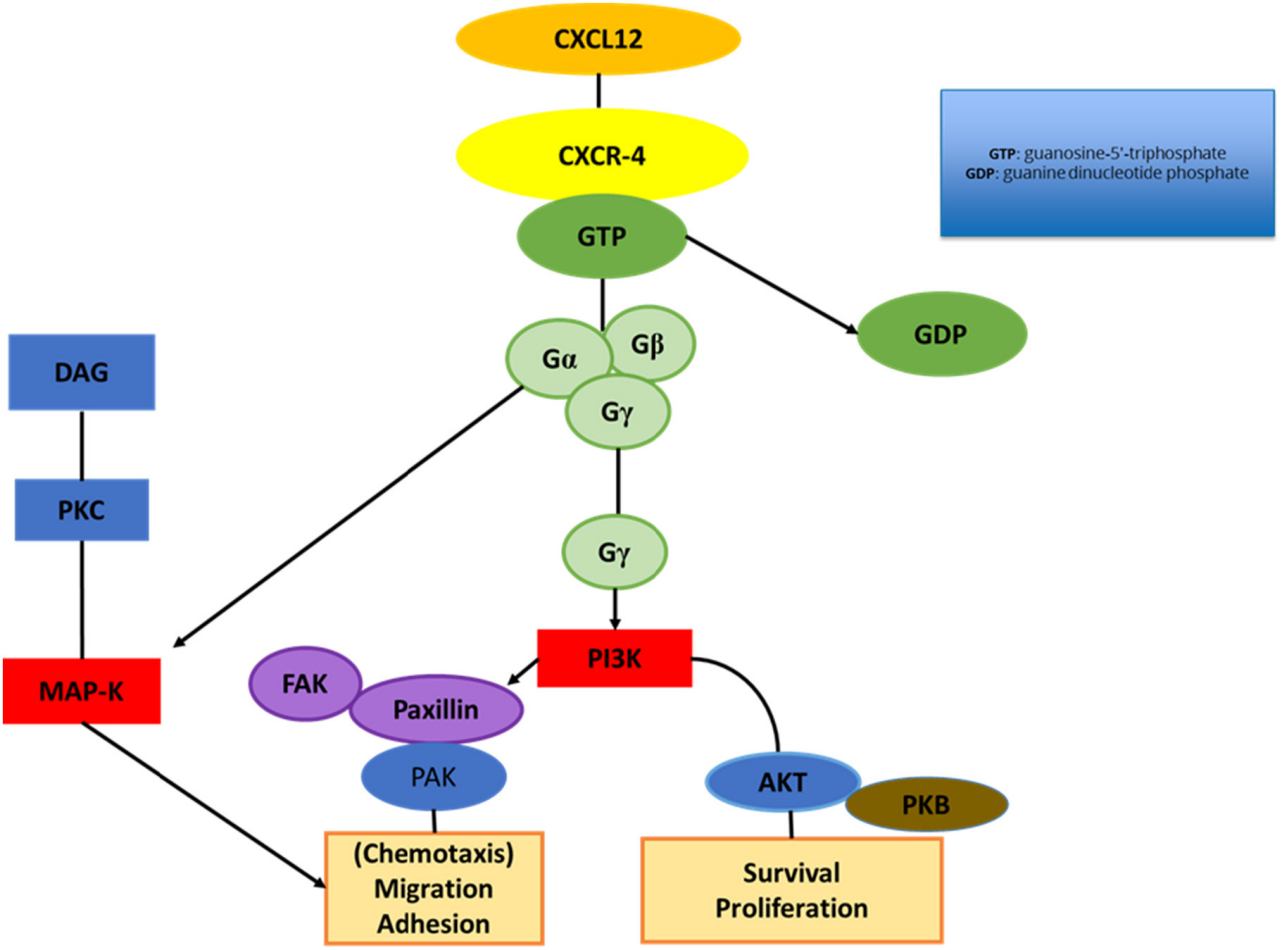
CXCR‐4 signalling pathway: MAP‐K and PI3K. A diagram indicating CXCR‐4 activation by the heterotrimeric G‐proteins (Gβ, Gα and Gγ) located on the intracellular section of the plasma membrane. Gα activates adenylate cyclase, which leads to MAP‐K activation. Gγ and Gβ activate the PI3K pathway, which leads to Akt stimulation resulting in tumour cell proliferation.^78^ Paxillin is a focal adhesion adapter protein. When paxillin is phosphorylated on tyrosine‐ and serine residue sites it recruits signalling molecules involved in cell migration.^81^ (Image was designed by Y.N Hlophe using Microsoft PowerPoint 2013; 2013 Microsoft Corporation).

CTCE‐9908 (CXCR‐4 antagonist) is a 17‐amino acid sequenced peptide consisting of a dimer of eight amino acid N‐terminal sequences,^75^, ^76^ that has shown to inhibit adhesion and growth of tumour cells.^77^ CTCE‐9908 is derived from human CXCL12,^78^, ^79^ and the NH_2_‐terminal sequence was modified on CTCE‐9908 to hinder the ligand CXCL12 binding to the receptor CXCR‐4.^80^ CTCE‐9908 therefore hinders receptor phosphorylation and the signalling pathways that would be activated (Figure 3).

The use of CXCR‐4 antagonists leads to receptor internalization and the dispersion of adhesion proteins resulting in a reduction in intercellular adhesion.^80^ Blocking CXCR‐4 with CTCE‐9908 can affect tumour cell metastasis if the blocking is conducted prior to the onset of metastasis.^77^ CTCE‐9908 inhibits CXCR‐4 phosphorylation by inhibiting the binding of the CXCL12 ligand, therefore inhibiting signalling pathways of migration. If tumour cells escape the inhibition of the CXCR‐4 antagonist, it still sensitizes the tumour cell, which renders it more receptive to therapies that can impact on tumour cell metastasis.^77^

## 4 ADHESION PROTEINS

### 4.1 Focal adhesion kinase

**FIGURE 4:**
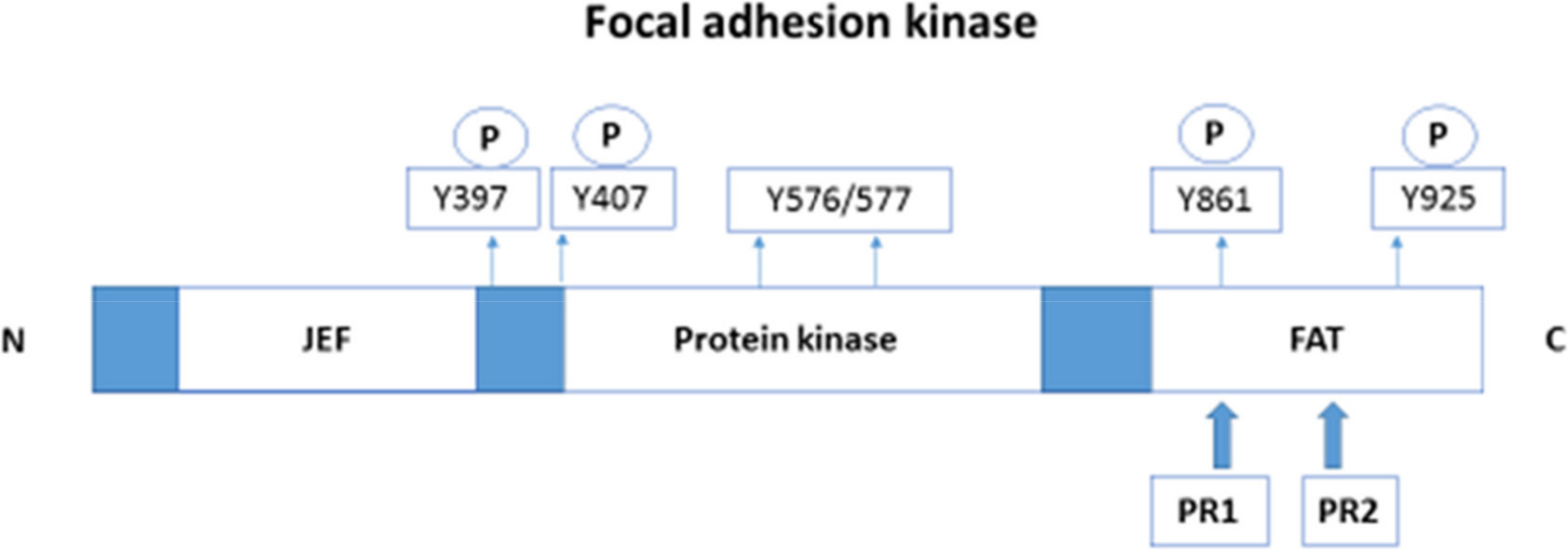
Focal adhesion kinase linear structure. The linear structure of FAK and tyrosine phosphorylation sites.^82^ The focal adhesion targeting (FAT) domain is important for adhesion‐dependent tyrosine phosphorylation and to contain integrin adhesion sites.^82^ The proline‐rich motifs (PR1 and PR2) facilitate interaction with the Src‐homology 3 domain.^82^ The majority of the N‐terminal comprises the JEF domain, although the function of the JEF domain is not well‐understood.^82^ (Image was designed by Y.N Hlophe using Microsoft PowerPoint 2013; 2013 Microsoft Corporation).

Focal adhesions host a protein tyrosine kinase that regulates signalling functions and controls cell behaviour as a result of integrin communication with the extracellular matrix.^82^ Studies have indicated FAK as a managing system in cell survival and motility. The signalling function of FAK is linked to the high phosphorylation rates as a result of integrin‐controlled action on the tyrosine (Tyr)‐397 site, allowing activity with Src‐homology domains. The Src family of kinases attracted to the Tyr‐397 site are responsible for phosphorylating two active FAK proteins, paxillin and Crk substrate (CAS), Rho families and GTPases and therefore play a role in cell motility (Figure 4).

The phosphorylation of FAK is crucial for the Src‐promoted downregulation of E‐cadherin observed in colon cancer cells (Avizienyte et al^83^) and the blockage of FAK showed a reduction in Src enhanced invasion.^84^ FAK is vital in the molecular mechanisms of E‐cadherin regulated cell–cell adhesions and integrin ‐ECM cross talk.^85^

### 4.2 Paxillin

Paxillin is a dominant focal adhesion that relays extracellular signals into intracellular responses facilitated by the interaction of integrins and the ECM.^81^ Paxillin is involved in the assembling of kinase, phosphatases, cofactors and oncoproteins involved in intracellular signalling cascades.^81^ The signalling cascades influence the actin cytoskeleton and focal adhesion adjustments, which affect cell adhesion and migration. Paxillin is detectable at the plasma membrane, cytoskeleton and nucleus.^81^ Paxillin does not promote enzymatic activity but provides docking sites for other proteins, which allow the assembly of multiprotein complexes.^81^

**FIGURE 5:**
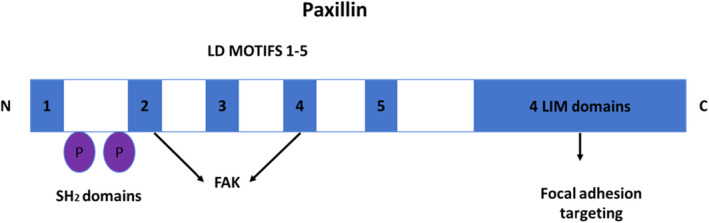
Paxillin linear structure. Linear paxillin structure indicating domains and tyrosine binding sites.^82^ Paxillin is a nonenzymatic docking protein with multiple binding domains.^82^ The 5 LD motifs are found at the N‐terminal and comprise eight residue leucine‐rich sequences.^82^ The LIM domains are double zinc‐finger motifs that form the C‐terminal.^82^ LIM 2 + 3 are important to recruit paxillin to focal adhesions.^82^ FAK relates with paxillin via LD motifs 2 + 4.^82^ (Image was designed by Y.N Hlophe using Microsoft PowerPoint 2013; 2013 Microsoft Corporation).

Paxillin is a tyrosine‐phosphorylated focal adhesion protein transformed by *v*‐SRC^82^ (kinase family of nonreceptor tyrosine kinases). Paxillin is not an enzyme but contains several protein‐interacting domains.^82^ The Lin 11, Is‐1 and Mec‐3 (LIM) protein domains (Figure 5) attract paxillin to FAK. Paxillin is activated in response to integrin adhesion‐mediated responses.^82^

### 4.3 Cadherin

Cadherins are a family of cell surface glycoproteins adhesion junctions that regulate cell–cell adhesion in a calcium‐dependent manner.^82^ Exterior domains of the cadherins bind neighbouring cells and cytoplasmic ends are bound to actomyosin cytoskeleton by means of catenins.^82^ Mechanical control between adhesion junctions and actomyosin cytoskeleton is regulated by Rho family of GTPases.^82^, ^86^ Studies have indicated that functional E‐cadherin in melanoma cells inhibits tumour growth.^82^ In melanomas, a cadherin switch occurs where the cells downregulate E‐cadherin and elevate concentrations of N‐cadherin resulting in melanoma cells detachment from the epidermis entering to penetrate vasculature.^87^

## 5 SIGNALLING PATHWAYS

### 5.1 Mitogen‐activated protein kinase and phosphatidylinositol 3‐kinase pathways

The MAP‐K pathway is a signalling cascade that plays a role in tumour progression.^88^ MAP‐K signals key molecules that sustain cell growth and proliferation.^88^ The cascade operates on growth factors and cytokine interaction with growth factor/cytokine receptor tyrosine kinases resulting in cellular intermediate transduction and eventually gene transcription/translation.^89^ Other pathways such as phosphoinositide‐3‐kinase/*v*‐Akt (P13k/Akt) interact with extracellular growth factors/cytokines that result in gene transcription/translation. MAP‐K mutations in solid tumours are prevalent in the RAS/RAF/MEK/ERK genes of the pathway.^90^ Melanoma is associated with ERK kinase mutations and this is visible in 3%–8% of melanoma cases^91^ The RAS gene mutation is associated with advancing tumours with poor prognosis and RAS activates MAP‐K/ P13K/Akt demonstrating the significance of the RAS in pathway changes observed in cancers.^92^ Alterations in the JNK2 gene impact signalling in the MAP‐K and P13K pathway.^93^ Due to their impact on tumour metastasis, it is important to determine the effect of the therapeutic targets on the MAP‐K and PI3K pathways and associated proteins.

### 5.2 Mitogen‐activated protein kinase and phosphatidylinositol 3‐kinase pathways linked to VEGFR‐3/VEGF‐C and CXCL12/CXCR‐4

**FIGURE 6:**
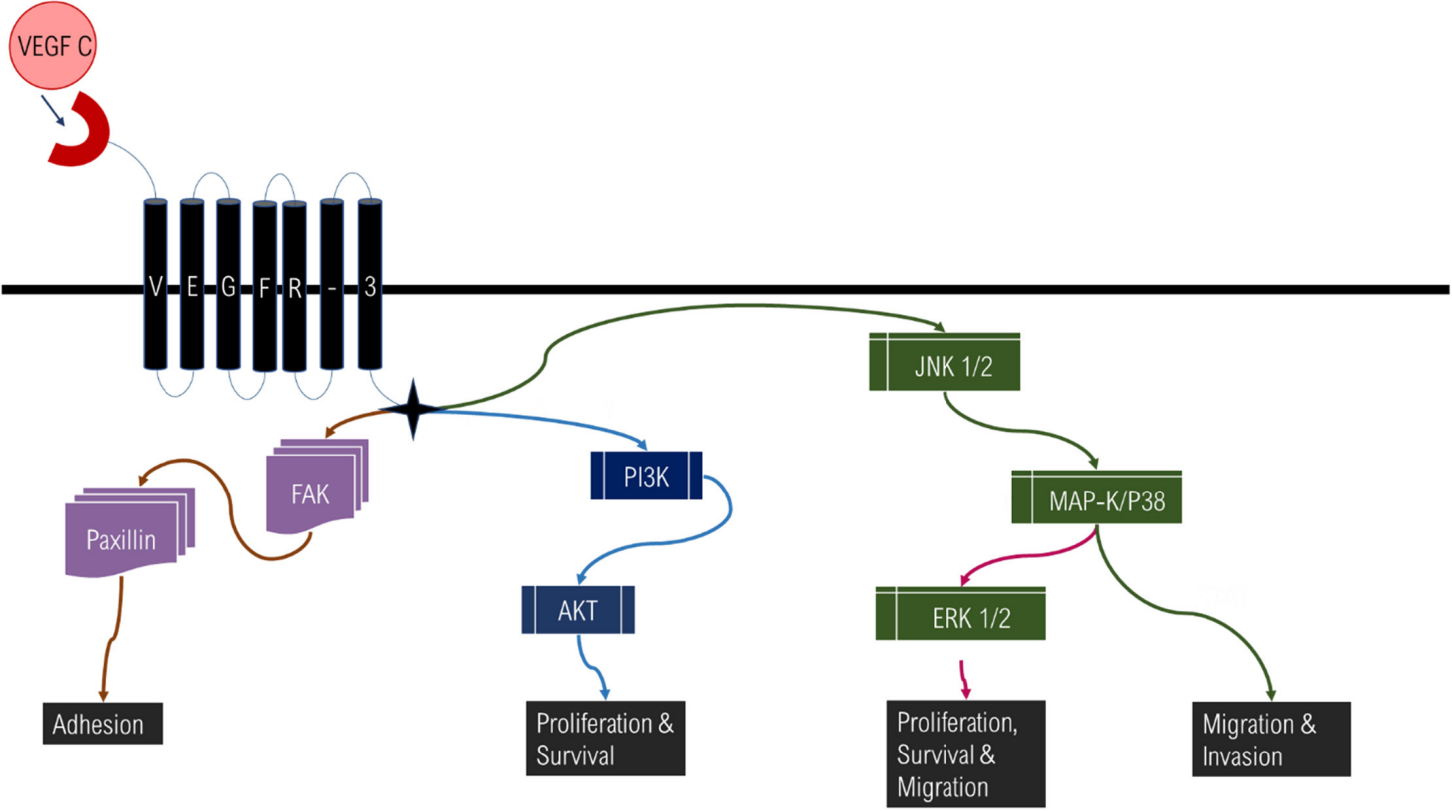
VEGFR‐3 signalling pathways: MAP‐K and PI3K. VEGF‐C binds VEGFR‐3 and activates JNK 1/2, MAP‐K/p38, extracellular signal‐regulated kinase (ERK) 1/2, PI3K/Akt pathways promoting migration, proliferation and survival of melanoma cells,^105^ ^.^ ^106^ Focal adhesion kinase (FAK) and paxillin are activated by growth factor signalling and facilitate tumour cell adhesion. (Image was designed by Y.N Hlophe and modified by Carolyn Nadasen using Microsoft PowerPoint 2013; 2013 Microsoft Corporation).

Research has shown that the VEGFR‐3/VEGF‐C gradient enhances invasion promoted by the Mitogen‐activated protein kinase/extracellular signal‐regulated kinases (MAP‐K/ERK) and the Phosphatidylinositol 3‐kinase/Akt strain transforming (PI3K/Akt) signalling pathways activated^4^, ^94^ as seen in Figure 6.

The activity of VEGF‐C/VEGFR‐3 is stimulated by protein kinase C (PKC).^95^ VEGF‐C has the ability to activate Jun N‐terminal Kinase (JNK) and Akt through activity with VEGFR‐3 in lymphatic endothelial cells (LECs).^95^ AKT is a downstream target of PI3K, which promotes cell survival and proliferation.^95^ The activation of VEGF‐C in LECs stimulates the phosphorylation of ERK1/2.^96^ The rapidly Accelerated Fibrosarcom/MAP‐K/ERK kinase (Raf/MEK) signalling through VEGF‐C activates ERK phosphorylation.^96^ VEGF‐C is known to phospholipase‐C‐γ 1 (PLC_ϒ_‐1) through VEGFR‐3 binding and PI3K in LECs.^96^ The activation of these pathways promotes proliferation, migration and survival of endothelial cells.^96^

Research conducted by Yeh et al linked the VEGFR‐3/VEGF‐C gradient to the MAP‐K signalling pathway (Figure 1) by studying yes‐associated protein 1 (YAP1), a transcriptional co‐activator that activates transcriptional activity leading to cell proliferation^97^ and Slug (human embryonic protein SNAI2) levels, a member of the snail family of transcriptional factors that inhibits E‐cadherin protein levels in melanoma cancer.^98^ Inhibition of VEGFR‐3/VEGF‐C activity inactivated YAP1 and Slug and therefore inhibited melanoma migration through MAP‐K signalling.^4^

Regulation of VEGF‐C gene transcription includes PI3K‐Akt (protein kinase‐B), signal‐regulated kinase 1/2, nuclear factor kappa‐light‐chain‐enhancer of activated B cells (NF_K_B) and p38 extracellular pathways.^99^ Regulators of VEGF‐C in melanoma include Wnt1 (wingless related integration site),^100^ epidermal growth factor^101^ and proto‐oncogene^102^ although the heterogeneity of VEG‐C protein levels in melanoma requires further understanding.^103^ The affinity of VEGF‐C to VEGFR‐3 and VEGFR‐2 allows the growth factor levels to promote tumour lymphangiogenesis and angiogenesis.^99^ In addition, the inhibition of lymphangiogenesis by blocking VEGFR‐3 in combination with VEGFR‐2 inhibits metastasis in mouse models.^104^

CXCR‐4 activates the PI3K pathway directly through activity at the Gβγ subunit of the receptor.^58^ PI3K activation leads to the activation of Akt (protein kinase‐B) promoting cancer cell survival and proliferation.^58^ PI3K activation through CXCR‐4 results in FAK activation, which then permits migratory activity in cancer cells (Figure 3).^58^, ^107^

**FIGURE 7:**
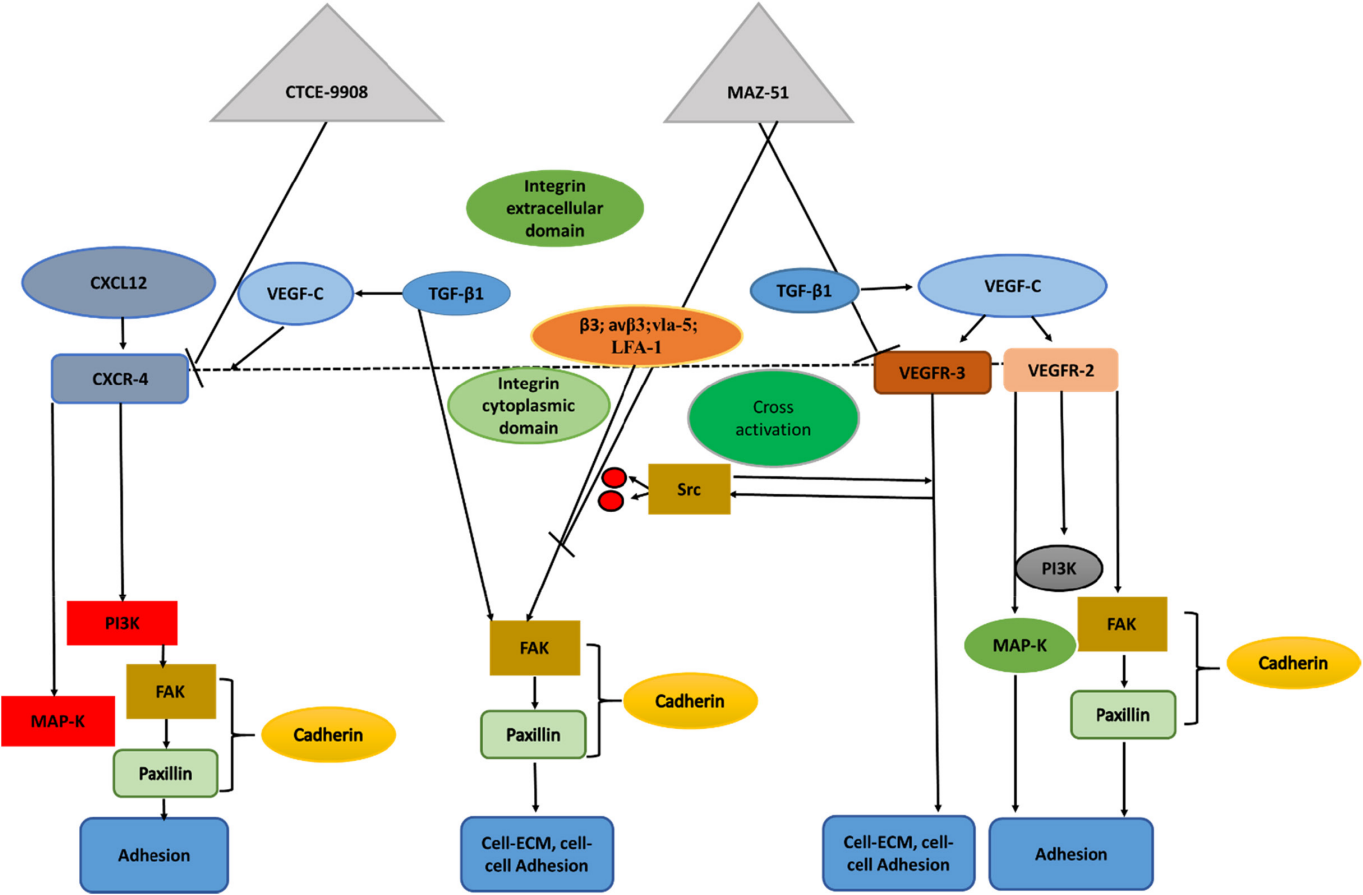
Ligand‐activated intracellular pathways promoting tumour cell adhesion. Activation of intracellular signalling pathways (MAP‐K and PI3K) promotes tumour cell adhesion once growth factor receptors (VEGFR‐2/3) and a chemokine receptor (CXCR‐4) are phosphorylated. TGF‐β1 ligand binding the two pair serine/threonine receptor activating receptor phosphorylation facilitates cell–cell and cell‐matrix adhesion by regulating cadherins through activation of focal adhesion kinase (FAK) and mediates paxillin upregulation. The activation of intracellular adhesion proteins (FAK, paxillin and cadherin) when ligands (VEGF‐C, TGF‐β1 and CXCL12) bind their specific receptors. Reduced expression of adhesion proteins will contribute to limiting the activity of the intracellular pathways that promote tumour cell adhesion. (Image was designed by Y.N Hlophe using Microsoft PowerPoint 2013; 2013 Microsoft CorporationUnited States of America).

CTCE‐9908 downregulates PI3‐K/Akt signalling targeting as seen in Figure 7 the CXCL12/CXCR‐4 axis in prostate cancer (PC‐3) cell line.^108^ In the PC‐3 xenograft model, CTCE‐9908 induced apoptosis and reduced nonspecific VEGF levels significantly.^17^, ^109^ CTCE‐9908 suppressed the expression of PI3‐K/Akt proteins and induced apoptosis in PC‐3 indicating the possible efficacy of CTCE‐9908 to hinder tumour cell adhesion. In a study conducted by Kim et al. B‐16 melanoma cells induced to express CXCR‐4 were intravenously injected into mice.^77^ Mice treated with CTCE‐9908 showed reduced protein levels of metastatic lung nodules in comparison to control mice. Treatment of mice with CTCE‐99O8 prior to intravenous injection of B‐16 cells expressing CXCR‐4,^77^ showed a greater percentage of reduced metastatic lung nodules indicating the significance of receptor site blocking to combat metastasis.^77^

## 6 DISCUSSION

In this review, the impact of CTCE‐9908 and MAZ‐51 on the CXCR‐4/CXCL12 and the VEGFR‐3/VEGF‐C gradient is addressed and the effects of MAZ‐51 and CTCE‐9908 on specific adhesion proteins of MAP‐K and PI3K signalling pathways are summarized.

Since CXCR‐4 is upregulated by VEGF‐C it forms an intricate link between the CXCR‐4/CXCL12 and VEGFR‐3/VEGF‐C gradients. In MDA‐MB231 cells, the inhibition of CXCL12 and VEGF‐C had an inhibitory effect on tumour lymphangiogenesis independent of VEGFR‐3.^50^ In addition, CTCE‐9908 and MAZ‐51 dissociate these gradients and disturb melanoma adhesion.

The inhibition of intracellular signalling pathways such as JNK/MAP‐K,^27^ PI3K^110^ and RAS^111^ have shown suppression of TGF‐β1 production directly indicating the role of the signalling pathways in TGF‐β1 production.^110^ TGF‐β11 observed in melanoma enhances growth factors such as VEGF, platelet‐derived growth factor receptor‐β, fibroblast growth factor receptor‐1, which promotes melanoma progression and collagens XV, XVI and XVIII and fibronectin important in melanoma adhesion.^28^

In melanoma, the downregulation of E‐cadherin and upregulation of N‐cadherin, which promotes tumour invasion^87^ indicates the need for cadherin levels to be monitored after exposure to MAZ‐51 and CTCE‐9908. If MAZ‐51 and CTCE‐9909 are able to reduce TGF‐β levels and downregulate N‐cadherin in melanoma cells, this may reduce the contributions of the two proteins on melanoma metastasis. Since melanoma cells express both the CXCR‐4 and VEGFR‐3 receptors, CXCR‐4 and VEGF may be regarded as quantifiable markers of tumour metastasis.^48^ Therapeutic strategies to inhibit the FAK signalling pathway are promising avenues to inhibit melanoma metastasis.^112^

## 7 CONCLUSION

The inhibitory effects of CTCE‐9908 and MAZ‐51 on VEGFR‐3 and CXCR4 protein expression show the potential to affect parameters of metastasis on melanoma cells by inhibiting cell–cell or cell‐ECM interactions by blocking downstream signalling molecules that impact on adhesion. Since integrin extracellular domains act as receptors for FAK^82^ and growth factors found in ECM of cancer cells,^113^ integrin protein expression levels should be assessed after combination, MAZ‐51 or CTCE‐9908 treatment to further understand the effects on melanoma adhesion properties. Future research using CTCE‐9908 and MAZ‐51 treatment, respectively, or combination treatment (CTCE‐9908‐MAZ‐51) are warranted to inhibit the determinination of tumour adhesion.

## AUTHOR CONTRIBUTIONS

**Yvette N Hlophe:** Conceptualization (lead); investigation (lead); project administration (equal). **A Joubert:** Project administration (equal); writing – review and editing (equal).

## CONFLICT OF INTEREST

The author(s) declared no potential conflicts of interest with respect to the compiling, authorship and/or publication of this manuscript.

## Data Availability

Data sharing is not applicable to this article as no new data were created or analyzed in this study.
